# Prevalence of *Eimeria* Spp. Among Goats in China: A Systematic Review and Meta-Analysis

**DOI:** 10.3389/fcimb.2022.806085

**Published:** 2022-03-02

**Authors:** Nai-Chao Diao, Bo Zhao, Yu Chen, Qi Wang, Zi-Yang Chen, Yang Yang, Yu-Han Sun, Jun-Feng Shi, Jian-Ming Li, Kun Shi, Qing-Long Gong, Rui Du

**Affiliations:** ^1^ College of Chinese Medicine Materials, Jilin Agricultural University, Changchun City, China; ^2^ College of Animal Science and Technology, Jilin Agricultural University, Changchun City, China; ^3^ College of Animal Science and Veterinary Medicine, Heilongjiang Bayi Agricultural University, Daqing City, China

**Keywords:** goat, *Eimeria*, prevalence, China, meta-analysis

## Abstract

*Eimeria* spp. infection can cause weight loss in goats, and severe cases can lead to the death of lambs, resulting in economic losses to the goat industry. To explore the pooled prevalence of *Eimeria* spp. in goats in China, we obtained 70 related publications from five databases and conducted a meta-analysis. In China, the combined prevalence of *Eimeria* spp. in goats was 78.7% (95% confidence interval (CI): 68.15–87.67). Among them, the most serious infections occurred in Northeast China (88.0%, 95% CI: 83.54–91.86). The main *Eimeria* species were *E. alijevi* (43.7%, 95% CI: 29.53–58.45), *E. arloingi* (49.7%, 95% CI: 34.83–64.49), *E. christenseni* (41.2%, 95% CI: 27.07–56.16), and *E. ninakohlyakimovae* (35.9%, 95% CI: 21.02–52.31). In the sampling year subgroup, 2006 or later presented a lower prevalence (75.3%, 95%CI: 58.72–88.72). In terms of age, the point estimate for young goats (≤ 1 year) was higher (89.9%, 95% CI: 80.82–96.48). The Float (NaCl) method showed the lowest prevalence of *Eimeria* spp. in goats (75.9%, 95%CI: 62.00–87.46). In the season subgroup, the highest prevalence was in summer (81.5%, 95%CI: 49.62–99.18). Female goats presented a higher prevalence of *Eimeria* spp. infection than male goats (70.7%, 95%CI: 27.90–98.96). The prevalence was lower in the intensive feeding model (77.4%, 95%CI: 66.56–86.67) and higher in free feeding goats (79.4%, 95%CI: 66.46–89.92). In addition, we also analyzed the potential relationship between geographical factors and the prevalence of *Eimeria* spp. infection in goats in China. Our findings suggested that *Eimeria* spp. infection in goats is widespread in China. Despite the overall downward trend, this infection cannot be ignored. We recommend that breeders use anticoccidial drugs to prevent and treat this disease, while improving the feeding conditions and managemental practices to reduce the economic losses caused by *Eimeria* infection to the goat industry.

## Introduction

In ruminants, coccidiosis is a parasitic disease caused by the *Eimeria* spp., which has a significant economic impact (Taylor et al., 2016; Keeton and Navarre, 2018; Bangoura and Bardsley, 2020). *Eimeria* spp. is distributed globally, and the infection rates can reach more than 90% in some areas (Cavalcante et al., 2012; Mohamaden et al., 2018; Juszczak et al., 2019). The main clinical feature of coccidiosis is diarrhea. Under conditions that promote *Eimeria* development, the accompanying clinical symptoms include low feed conversion rate, weight loss, and lethargy (Foreyt, 1990).

*Eimeria* has a high degree of host specificity, with different species of *Eimeria* in goats and sheep, among which 13 species of *Eimeria* are currently recognized to infect goats (Lotze et al., 1961; Bangoura and Bardsley, 2020). Among the 13 species of *Eimeria*, *E. ninakohlyakimovae* and *E. arloingi* are considered to be more pathogenic.

China is one of the most important agricultural countries in the world, and since the late, 1980s, China has become the country with the largest number of goats (Liu et al., 2018; Wang et al., 2020). Goat meat and mutton production reached 4.68 million tons in, 2017 (Liu et al., 2018). *Eimeria* spp. infection affects the health of goats, thereby affecting their production profits. Consequently, we conducted a systematic review and meta-analysis of the prevalence of *Eimeria* spp. in goats in China, taking into account sampling year, age, species, detection methods, feeding model, season, presence of diarrhea, regions, and quality level, to determine the factors which affect *Eimeria* prevalence in goats. Furthermore, geographical factors (longitude, latitude, and altitude) and climatic factors (annual temperature, maximum and minimum temperature, rainfall, and humidity) were analyzed in our meta-analysis, which might be potential factors influencing *Eimeria* infection in goats. Exploration of the prevalence and geographical distribution of *Eimeria* in goats in China along with the identification of the predisposing factors might highlight weak points and accelerate the future eradication of *Eimeria*.

## Material and Methods

### Search Strategy

Our research was performed according to the Preferred Reporting Items for Systematic Review and Meta-Analysis (PRISMA) protocols (**Table S1**) (Moher et al., 2015). To obtain the maximum number of publications, we searched in five databases (China national knowledge internet (CNKI), VIP databases, Wan Fang databases, PubMed, and ScienceDirect). In PubMed, we used the MeSH index to determine the following subject terms: “Goats”, “*Eimeria*”, “China”. In MeSH Terms, the free words obtained by goats were: “Goat”, “Capra” and “Capras”. The free words obtained by *Eimeria* were: “*Eimerias*”, “Coccidia” and “Coccidias”. China’s free words were: “People’s Republic of China”, “Mainland China”, “Manchuria”, “Sinkiang”, and “Inner Mongolia”. We used the “OR” combination between subject words and free words. Finally, the search formula we established was as follows: (“Eimeria”[Mesh] OR Eimerias OR Coccidia OR Coccidias) AND (“Goats”[Mesh] OR Goat OR Capra OR Capras) AND (“China”[Mesh] OR People’s Republic of China OR Mainland China OR Manchuria OR Sinkiang OR Inner Mongolia). In ScienceDirect, we searched using the keywords “Goats”, “*Eimeria*” and “China”, and the title, abstract, and keywords must include “China”. In the three Chinese database (CNKI, VIP and WanFang), the search query we chose was “Goat” and “*Eimeria*” in Chinese, and synonym expansion and fuzzy search were enabled. We conducted a final search on October 9, 2021.

### Inclusion and Exclusion Criteria

The literature information was processed using EndNote X9.3.2 for summarization (Wang et al., 2021). After excluding duplicate articles, three systematically trained researchers reviewed the titles and abstracts of all the articles and conducted the preliminary screening. To ensure the quality of the included articles, we have established the following inclusion criteria, based on the premise that full text and original research could be obtained:

(1) The study purpose was to examine the prevalence of *Eimeria* among goats in China;
(2) The study was published in English or Chinese;
(3) One sample was taken from each goat (not mixed samples);
The exclusion criteria comprised:
(1) Articles with incorrect data;
(2) Articles reporting the same data;
(3) Review articles;
(4) Articles dealing with other parasitic disease prevalence surveys;
(5) Articles reporting data for other hosts.

### Data Extraction

All the articles were distributed to three trained reviewers (BZ, ZYC, and YY) for review. The extracted data included: First author, publication year, sampling year, geographical factors of sampling location (latitude and longitude, rainfall, annual average temperature, annual minimum temperature, annual maximum temperature), detection method, sex, age, breeding method, and season. In the process of selecting the articles, we neither contacted the authors of the articles to obtain more research information, nor included unpublished data (**Table S2**). Any doubts and uncertainties about the data of the included articles were processed uniformly after evaluation by the major reviewer (QLG, the methodology provider for this meta-analysis).

### Quality Assessment

The quality of the included articles was evaluated by means of scoring (Guyatt et al., 2008; Gong et al., 2021). The specific method was as follows: Each of the below mentioned five points was counted as one point: (1) Whether there is a sampling time, (2) whether the sampling method is described in detail, (3) whether random sampling was used, (4) whether there was a detection method, and (5) whether it included 3 subgroups or more. According to the score of each article, it was assigned to the corresponding level. There were three levels: 0–1 point, 2–3 points and 4–5 points (**Table S3**). The data gleaned from the included studies were summarized and edited using Microsoft Excel (version 16.32; Microsoft Corp., Redmond, WA, USA).

### Statistical Analysis

We used the “meta” package in the R software to perform this meta-analysis (“R core team, version 4.0.0; “R: A language and environment for statistical computing”, R core team, 2018) (Wang, 2018). According to the description of the conversion rate in a previous study, we used the Freeman-Tukey double arcsine transformation (named “PFT” in the meta package) to perform conversion to conform to the normal distribution (**Table 1** and **Table S4**) (Li et al., 2020). The combined estimates included in the study were described using forest plots. The heterogeneity in the prevalence meta-analysis is usually very large, therefore, we made a judgment in advance and used a random-effect model to analyze the overall prevalence (including subgroups). For the differences caused by the heterogeneity of the included studies, Cochran’s Q statistics and Higgin’s statistics were used for evaluation. In the funnel diagram, the symmetry of the figure was judged subjectively. If the dots in the funnel plot were symmetrically distributed on both sides of the symmetry line, there was no publication bias, if they were asymmetric, there was a publication bias in the included studies. At the same time, we used sensitivity analysis and trimming and filling analysis to evaluate the reliability of the articles and used Egger’s test and funnel plots to estimate the heterogeneity in the included studies (Li et al., 2020; Gong et al., 2021).

**Table 1:** Normal distribution test for the normal rate and the different conversion of the normal rate.

Conversion form	*W*	*P*
PRAW	0.81473	6.021e-08
PLN	0.5427	1.968e-13
PLOGIT	NaN	NA
PAS	0.89765	3.134e-05
PFT	0.88739	1.275e-05

“PRAW”, original rate; “PLN”, logarithmic conversion; “PLOGIT”, logit transformation; “PAS”, arcsine transformation; “PFT”, double-arcsine transformation, NA, No answer; NaN, Not a number.

To track the potential sources of heterogeneity in our study, we performed subgroup analysis and univariate meta-regression (Wang et al., 2017). The potential factors included geographical area (Central China, Eastern China, Northern China, Northeastern China, Northwestern China, Southern China, and Southwestern China); sampling year (before, 2006 and, 2006 or later); detection method (Float (NaCl), Float (C_12_H_22_O_11_), and others); feeding model (free range vs. intensive); age (≤1 year and > 1 year); sex (male and female); season (spring, summer, autumn, and winter), score level (2–3 points vs. others). We further extracted geographical factors based on the sampling location using data obtained from the National Meteorological Information Center of China Meteorological Administration for subgroup analysis and univariate meta-regression to track the source of heterogeneity. We inquired about the latitude, longitude, precipitation, annual average temperature, annual average humidity and altitude of each sample source, and divided each factor into different intervals, including latitude (20–30°, 30–35°, 35–40°, and 40–50°), longitude (80–105°, 105*–*110°, 110*–*120°, 120*–*125°), precipitation (0*–*400 mm, 400*–*800 mm, and 800*–*2000 mm), annual average temperature (-5–10°C, 10–15°C, and 15*–*20°C), annual average humidity (30*–*60%, 60*–*70%, 70*–*80%, and 80*–*100%), and altitude (4*–*100 m, 100*–*1500 m, and, 1500*–*5000 m). Our meta-analysis was not registered in the Cochrane database. The code in R software for this study is provided in **Table S5**.

## Results

### Study Characteristics

**Figure 1:**
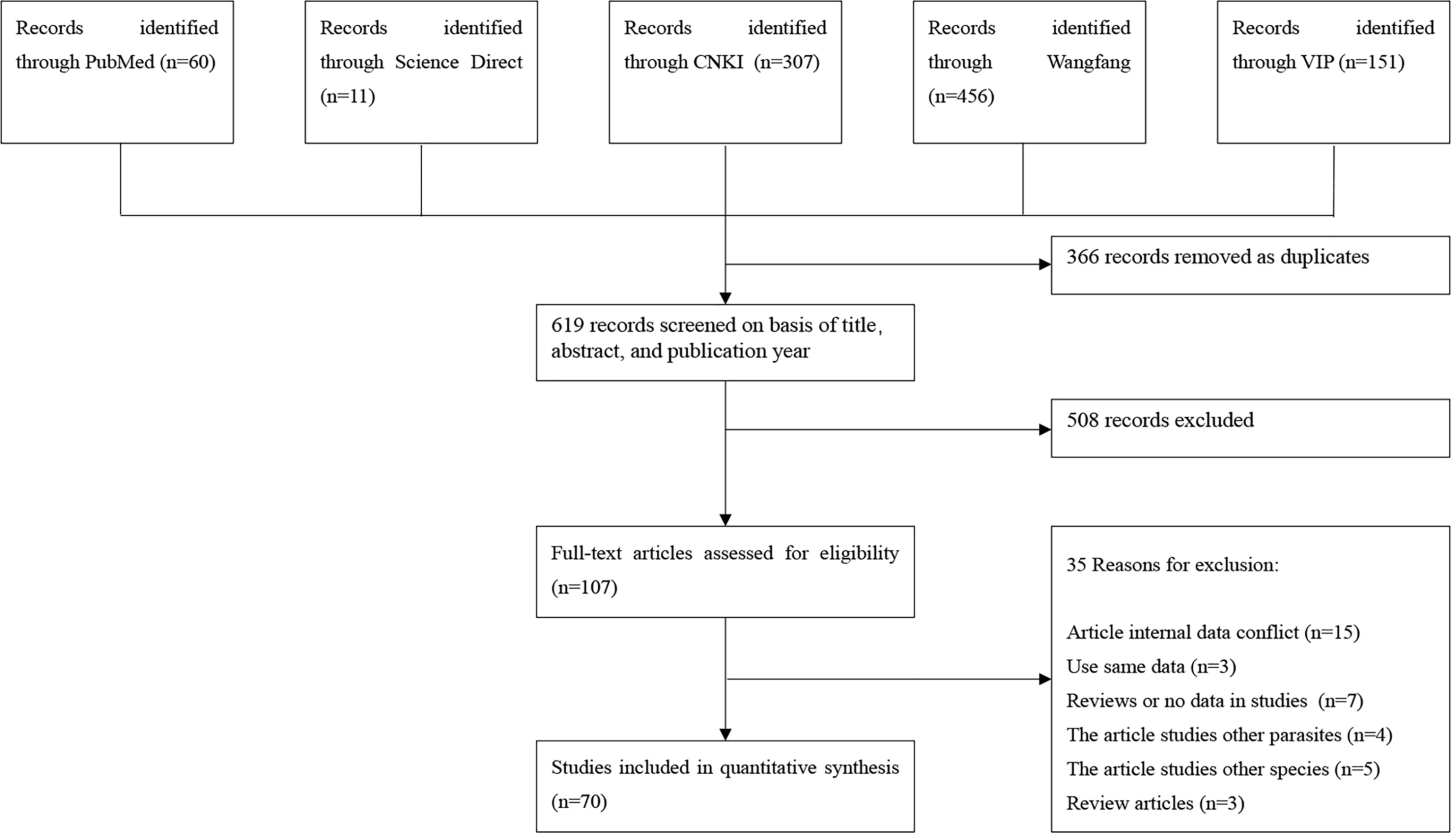
Flow diagram of eligible studies for searching and selecting.

According to our inclusion criteria, 985 articles were collected from five databases, and 70 studies were finally included to build this meta-analysis (**Figure 1**). A total of 40 studies scored 4–5 points, 25 studies scored 2–3 points, and only 5 studies scored 0–1 point (**Tables S2**, **S3**).

### Pooled Estimates and Heterogeneity Analyses

**Figure 2:**
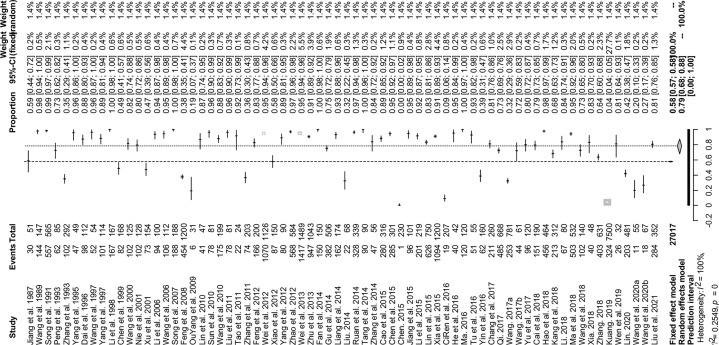
Forest plot of prevalence of *Eimeria* spp. in goats amongst studies conducted in China. The length of the horizontal line represents the 95% CI; the diamond represents the summarized effect.

**Figure 3:**
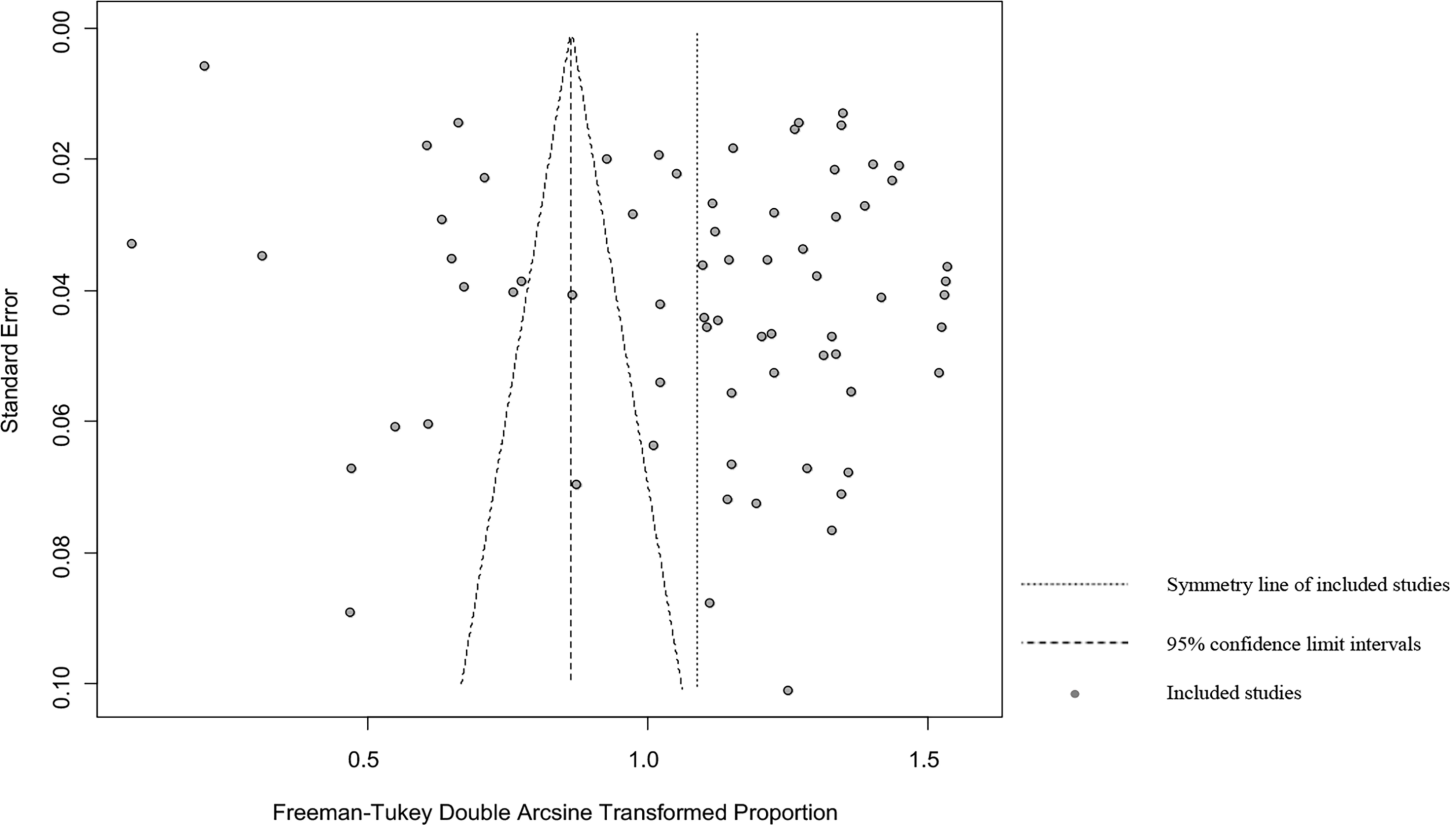
Funnel plot with pseudo 95% confidence interval limits for the examination of publication bias.

**Figure 4:**
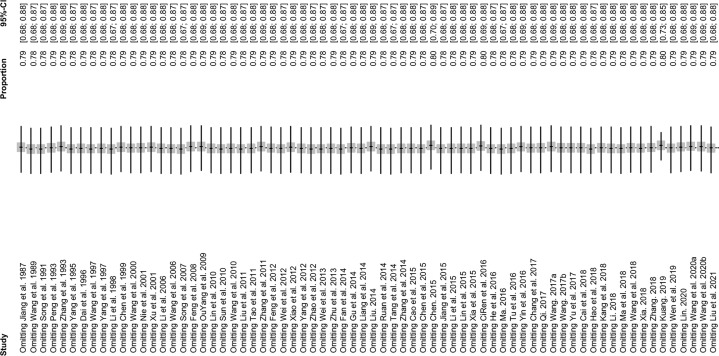
Sensitivity analysis. After removing one study at a time, the remaining studies were re-combined using a random-effects model to verify the impact of a single study on the overall results.

The forest plots showed there was a high heterogeneity in the included studies (*I^2^* = 99.7%, *P* < 0.01; **Figure 2**). In the funnel plot, we observed asymmetry, which indicated that there was a publication bias in our meta-analysis (**Figure 3**). The Egger’s test showed the same result as the funnel chart (*P* < 0.05; **Figure S1**, **Table S6**). The trim and fill analysis showed that the number of added studies was 33, indicating that there was publication bias or small sample bias in our included studies (**Figure S2**). Sensitivity analysis verified the reliability of the results, and excluding any one study had little effect on the overall quality of the meta-analysis (**Figure 4**). We also provided the funnel plot for each subgroup to determine whether there was a publication bias or small-sample bias (**Figures S3**–**S10**).

### Meta-Analysis

**Figure 5:**
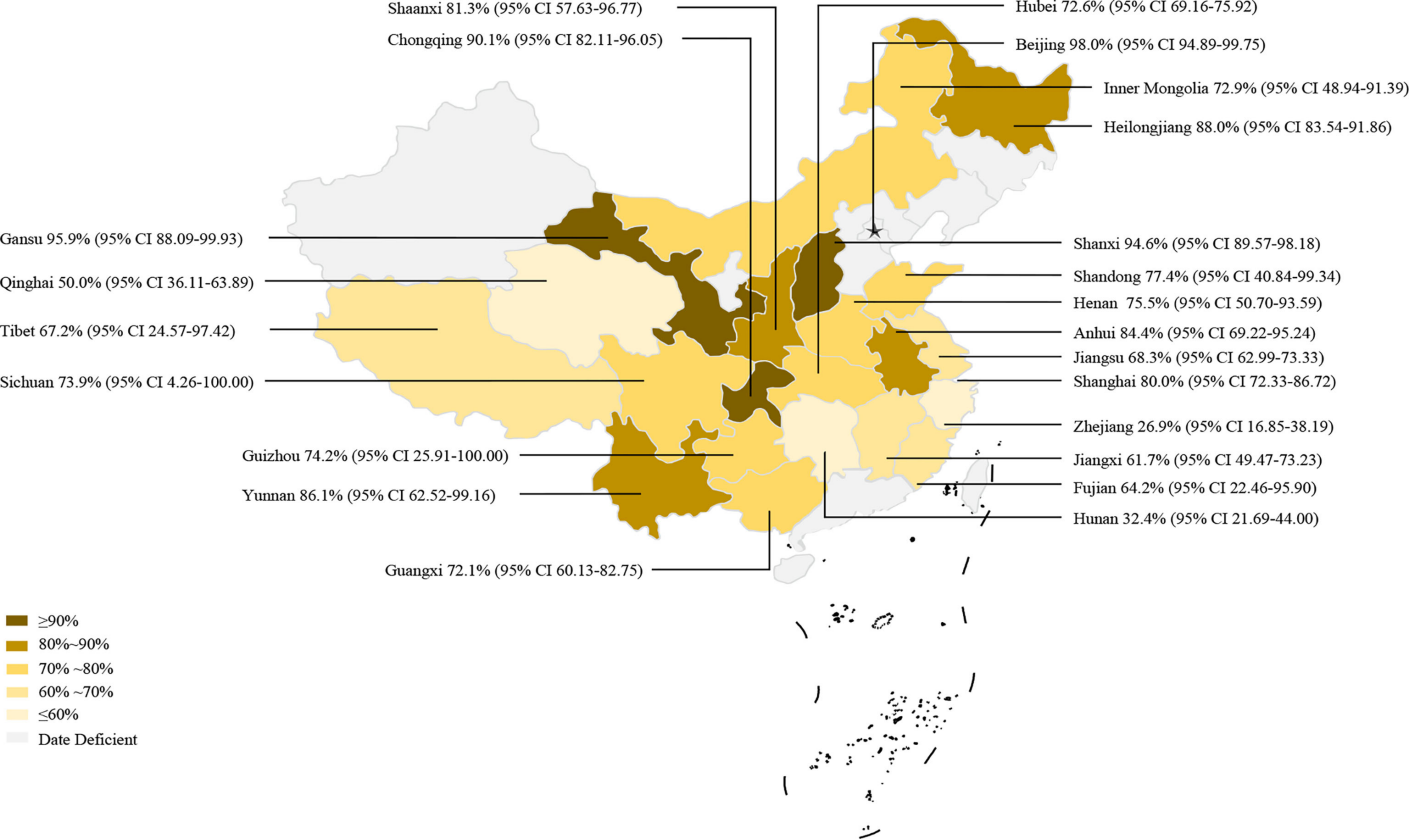
Map of *Eimeria* spp. in goats amongst studies conducted in China.

In the 70 selected studies, the pooled prevalence of *Eimeria* spp. infection of goats in China was 78.7% (95% CI: 68.15–87.67; 15,635/27,388) (**Table 2**). In terms of regions, the highest prevalence was in the Northeast (88.0%, 95% CI: 83.54–91.86; 216/246), and the lowest prevalence was in Central China (70.9%, 95% CI: 50.57–87.63; 2,559/3,255). At the provincial level, except for Zhejiang (26.9%, 95% CI: 16.85–38.19; 18/67), in the other provinces, the prevalence was above 50% (**Figure 5** and **Table 3**). In the sampling year subgroup, we found that the prevalence of *Eimeria* spp. in goats in China showed a downward trend. In terms of age, the estimated prevalence of goats ≤ 1 year old (89.9%, 95%CI: 80.82–96.48; 3,153/3,677) was higher than that at > 1 year (82.2%; 95% CI: 73.97–89.15; 3,689/4,357). In the sex subgroup, male goats (70.7%, 95%CI: 27.9–98.96, 120/211) had a lower *Eimeria* spp. prevalence than female (89.9%, 95%CI: 70.86–99.78, 800/897). The estimated pooled prevalence of *Eimeria* spp. detected using the Float (NaCl) method was 75.9% (95% CI: 62.00–87.46; 8,220/18,613), which was lower than that using the Float (C_12_H_22_O_11_) method (85.7%; 95% CI: 75.25–93.67; 5,867/7,016). Among seasons *Eimeria* spp. were more prevalent in summer (81.5%, 95%CI: 46.52–99.73; 1,625/3,648). In the feeding model subgroup, the estimated prevalence among free range goats (79.4%, 95%CI: 66.46–89.92; 2,295/2,940) was higher than in intensively farmed goats (77.4%, 95%CI: 66.56–86.67, 6,326/8,521) (**Table 2**). The articles which got 2–3 score level (69.0%, 95%CI: 57.46–79.55; 3,682/5,980) have reached the lowest prevalence among the three score levels.

**Table 2:** Pooled prevalence of *Eimeria* spp. infection in goats in China.

		No. studies	No. examined	No. positive	% (95% CI*)	Heterogeneity	*P*-value	Univariate meta-regression		
						*χ* ^2^		*I^2^* (%)	*P*-value	Coefficient (95% CI)
Region*									0.584	-0.099 (-0.455 to 0.256)
	Central China	9	3255	2559	70.9% (50.57-87.63)	1066.20	< 0.01	99.2%	Reference	
	Eastern China	24	4935	3384	79.8% (69.78-88.21)	1331.54	< 0.01	98.3%		
	Northern China	5	1081	777	78.4% (57.25-93.78)	175.84	< 0.01	97.7%		
	Northeastern China	2	246	216	88.0% (83.54-91.86)	0.06	0.81	0.00%		
	Northwestern China	12	2985	1996	80.7% (59.36-95.42)	1562.79	0.00	99.3%		
	Southern China	1	61	44	72.1% (60.13-82.75)	0.00	–	–		
	Southwestern China	21	12714	4727	78.4% (50.75-96.62)	14870.98	0.00	99.9%		
**Sampling year**									0.508	0.105 (-0.206 to 0.416)
	Before, 2006	16	2794	2238	83.9% (70.48-93.84)	910.51	< 0.01	98.4%	Reference	
	2006 or later	38	20728	10823	75.3% (58.72-88.72)	21371.00	0.00	99.8%		
Detection methods*									0.343	-0.129 (-0.396 to 0.138)
	Float (NaCl)	50	19189	8774	75.9% (62.00-87.46)	18390.77	0.00	99.7%	Reference	
	Float (C_12_H_22_O_11_)	14	7016	5867	85.7% (75.25-93.67)	1537.52	< 0.01	98.2%		
	Others	2	218	197	87.7% (27.78-100.00)	68.59	< 0.01	98.5%		
**Feeding model**									0.829	0.022 (-0.180 to 0.225)
	Free range	16	2940	2295	79.4% (66.46-89.92)	884.77	< 0.01	98.3%	Reference	
	Intensive	33	8521	6326	77.4% (66.56-86.67)	3966.04	0.00	99.2%		
**Age**									0.165	-0.111 (-0.268 to 0.046)
	> 1 year	23	4357	3689	82.2% (73.97-89.15)	800.04	< 0.01	97.3%		
	≤ 1 year	23	3677	3153	89.9% (80.82-96.48)	1240.90	< 0.01	98.2	Reference	
**Sex**									0.258	-0.246 (-0.672 to 0.180)
	Female	6	897	800	89.9% (70.86-99.78)	168.80	< 0.01	97.0%		
	Male	4	211	120	70.7% (27.90- 98.96)	115.08	< 0.01	97.4%	Reference	
Season*									0.324	0.181 (-0.178 to 0.541)
	Spring	12	3116	791	60.5% (32.14-85.58)	1946.74	0.00	99.4%		
	Summer	10	3648	1625	81.5% (46.52-99.73)	3687.38	0.00	99.8%	Reference	
	Autumn	10	4297	1580	70.5% (41.19-92.73)	2929.92	0.00	99.7%		
	Winter	5	2210	284	67.8% (8.79- 100.00)	1322.86	< 0.01	99.7%		
**Score level**									0.188	-0.170 (-0.424 to 0.083)
	4–5	40	20130	10805	82.9% (67.49-94.10)	22252.61	0.00	99.8%		
	2–3	25	5980	3682	69.0% (57.46-79.55)	1991.91	0.00	98.8%	Reference	
	0–1	5	907	829	88.8% (80.67-94.99)	30.53	< 0.01	86.9%		
**Total**		70	27017	15316	78.7% (68.15-87.67)	25056.86	0.00	99.7%		

CI*, Confidence interval;

NA*, not applicable;

Region*: Northern China: Beijing; Northwestern China: Shanxi, Gansu, Inner Mongolia, Shaanxi, Qinghai; Southwestern China: Chongqing, Guizhou, Sichuan, Tibet, Yunnan; Northeastern China: Heilongjiang; Central China: Henan; Eastern China: Fujian, Jiangsu, Anhui, Zhejiang, Shandong; Southern China: Hubei, Guangxi.

Detection methods*: Float (NaCl): Saturated Salt Water Floatation Method; Float (C_12_H_22_O_11_): Saturated Sucrose Solution Floatation Method; Others: Stauer’s Method, Saturated Magnesium Sulfate Solution as Test Tube Floatation Method.

Season*: Spring: Mar to May; Summer: Jun to Aug; Autumn: Sep to Nov; Winter: Dec to Feb.

**Table 3:** Pooled prevalence of *Eimeria* spp. by provincial in China.

Province	No. studies	Region	No. tested	No. positive	% Prevalence	% (95% CI)
Anhui	11	East China	1977	1261	83.0%	64.96-95.53
Beijing	1	North China	147	144	98.0%	94.89-99.75
Chongqing	4	Southwest China	660	600	90.1%	82.11-96.05
Fujian	2	East China	1231	829	64.2%	22.46-95.90
Gansu	1	Northwest China	49	47	95.9%	88.09-99.93
Guangxi	1	South China	61	44	72.1%	60.13-82.75
Guizhou	5	Southwest China	1341	1009	74.2%	25.91-100.00
Heilongjiang	2	Northeast China	246	216	88.0%	83.54-91.86
Henan	7	Central China	2519	2052	75.5%	50.70-93.59
Hubei	1	Central China	668	485	72.6%	69.16-75.92
Hunan	1	Central China	68	22	32.4%	21.69-44.00
Inner Mongolia	3	North China	822	527	72.9%	48.94-91.39
Jiangsu	7	East China	720	534	68.3%	62.99-73.33
Jiangxi	1	East China	312	213	61.7%	49.47-73.23
Qinghai	1	Northwest China	50	25	50.0%	36.11-63.89
Shaanxi	10	Northwest China	2886	1924	81.3%	57.63-96.77
Shandong	3	East China	137	114	77.4%	40.84-99.34
Shanghai	1	East China	120	96	80.0%	72.33-86.72
Shanxi	1	North China	112	106	94.6%	89.57-98.18
Sichuan	4	Southwest China	8121	923	73.9%	4.26-100.00
Tibet	4	Southwest China	1715	1364	67.2%	24.57-97.42
Yunnan	4	Southwest China	877	831	86.1%	62.52-99.16
Zhejiang	1	East China	67	18	26.9%	16.85-38.19
Total	76		24906	13384	77.2%	66.62-86.27

Twelve species of *Eimeria* were found in goats of China. Among them, *E. arloingi* had the highest prevalence (49.7%, 95%CI: 34.83–64.49; 2,417/5,595) (**Table 4**). On the basis of our calculated prevalence of *Eimeria* spp. in goats in China, we used data from, 2018 data of the *Chinese Animal Husbandry and Veterinary Yearbook* report to calculate that there were 108,792,519 (94,277,634–121,233,849) cases of *Eimeria* infection in goats in China (**Table S7**).

**Table 4:** Estimates of *Eimeria* spp. prevalence in goats in China.

	No.studies	No. tested	No. positive	Prevalence of infection
*E. alijevi*	16	5365	1791	43.7% (29.53-58.45)
*E. apsheronica*	12	4463	276	9.71% (5.21-15.35)
*E. arloingi*	17	5595	2417	49.7% (34.83-64.49)
*E. caprina*	16	5365	1505	36.6% (21.18-53.44)
*E. caprovina*	8	3883	400	12.1% (4.92-21.6)
*E. christenseni*	18	5984	2003	41.2% (27.07-56.16)
*E. hirci*	13	4896	1515	37.8% (22.95-53.88)
*E. jolchijevi*	14	4976	847	16.3% (8.09-26.49)
*E. kocharli*	2	1749	99	6.9% (3.01-12.16)
*E. ninakohlyakimovae*	12	3937	731	35.9% (21.02-52.31)
*E. pallida*	3	1803	160	13.6% (0.00-57.71)
*E. punctata*	5	2397	48	3.0% (0.40-7.34)

We also conducted a subgroup analysis of geographical factors. The prevalence was highest when the longitude is 80–105° (84.6%, 95%CI: 74.84–92.34; 2,270/2,519), and the same was true for latitudes 30*–*35° (80.5%, 95%CI: 68.94–90.00, 4,554/6,437), precipitation > 800–2000 mm (79.8%, 95%CI: 64.86–91.47; 3,827/3,094), the annual average temperature -5–10°(84.8%, 95%CI: 78.36–90.29; 2,088/2,385), humidity 80–100% (95.7%, 95%CI: 93.14–97.72; 629/663), and altitude above, 1500–5000 m (81.2%, 95%CI: 72.23–88.79; 2,600/2,325) (**Table 5**).

**Table 5:** Subgroup analysis of the prevalence of *Eimeria* spp. according to geographic location and climatic variables.

		No. studies	No. examined	No. positive	% (95% CI*)	Heterogeneity	*P*-value	Univariate meta-regression		
						*χ* ^2^		*I^2^* (%)	*P*-value	Coefficient (95% CI)
**Latitude**									0.523	-0.057 (-0.121 to 0.234)
	20–30°	20	4,954	3,891	76.1% (61.25–88.32)	2329.12	0.00	99.2		
	30*–*35°	28	6347	4554	80.5% (68.94–90.00)	2901.06	0.00	99.1	Reference	
	35*–*40°	11	2,774	1,645	76.5% (59.07–90.28)	795.66	<0.01	98.7		
	40–50°	2	250	197	68.2% (21.29–99.19)	40.18	<0.01	97.5		
**Longitude**									0.533	0.071 (-0.152 to 0.294)
	80–105°	10	2,519	2,270	84.6% (74.84–92.34)	258.58	<0.01	96.5	Reference	
	105*–*110°	22	5,404	3,766	79.7% (64.91–91.31)	3009.06	0.00	99.3		
	110*–*120°	26	5196	3597	77.4% (66.03–87.06)	2011.65	0.00	98.8		
	120–125°	5	578	454	75.2% (55.63–90.63)	98.02	<0.01	95.9		
**Precipitation** (mm)									0.474	-0.088 (-0.330 to 0.154)
	0–400	10	3,131	2,131	71.4% (49.37–89.09)	1257.90	<0.01	99.3	Reference	
	400*–*800	14	2742	2205	77.8% (65.09–88.40)	669.85	<0.01	98.1		
	800-2000	21	3827	3094	79.8% (64.86–91.47)	2143.59	0.00	99.1		
**Temperature** (°C)									0.277	0.128 (-0.103 to 0.360)
	-5–10	12	2,385	2,088	84.8% (78.36–90.29)	133.05	<0.01	91.7	Reference	
	10–15	11	2134	1538	72.0% (44.08–93.08)	1654.60	0.00	99.4		
	15–20	23	5181	3804	75.0% (61.20–86.55)	2374.38	0.00	99.1		
**Humidity** (%)									0.139	0.303 (-0.098 to 0.705)
	30–60	12	3,658	3,214	82.6% (72.24–90.96)	546.34	<0.01	98.0		
	60*–*70	17	1,948	1,494	76.1% (62.91–0.87)	609.19	<0.01	97.4		
	70*–*80	19	3431	2093	76.7% (58.87–90.70)	2271.35	0.00	99.2		
	80–100%	3	663	629	95.7% (93.14-97.72)	2.51	0.29	20.2	Reference	
**Altitude** (m)									0.817	-0.020 (-0.186 to 0.146)
	4–100	26	3441	2391	79.2% (67.46–88.92)	1484.36	<0.01	98.3		
	100*–*1500	30	7636	5,371	77.7% (65.64–87.73)	3878.01	0.00	99.3	Reference	
	1500–5000	9	2,600	2325	81.2% (72.23–88.79)	179.51	<0.01	95.5		

CI*, Confidence interval;

NA*, Not applicable.

## Discussion

Coccidiosis caused by *Eimeria* spp. is one of the most common intestinal diseases in goats (Ruiz et al., 2006). Whether there is a clinical infection, or the goat is in a subclinical state, it will cause economic losses (Zhao et al., 2012; Keeton and Navarre, 2018). Therefore, we conducted a meta-analysis of *Eimeria* spp. infection of goats in China. In, 2006, the China’s Ministry of Agriculture issued the “Parasitic Disease Control Plan (2006–2016)”, which was subsequently extended to, 2021, in order to further strengthen the prevention and control of parasites. Therefore, we used, 2006 as the boundary to analyze the changes in point estimates of Chinese goat coccidiosis. We found that the point estimate of goat coccidiosis in, 2006 or latter decreased. Before, 2006, the main goal of Chinese animal husbandry was to increase production rapidly and optimize the industrial structure (Wang, 2015), thus ignoring the environmental pollution caused by the wastewater and feces from goat breeding farms. The implementation of disease control policies has brought positive results due to changes in managemental aspects leading to a decline in the prevalence of goat coccidiosis. Notably, the differences between year subgroups were not significant. This might have been because we only had 2,794 samples prior to, 2006. Therefore, further studies are needed to demonstrate whether there is a downward trend in goat coccidiosis or not.

In China, goat coccidiosis is widespread, and infections were found in all areas. According to the analysis of the geographical subgroups, we found the highest point estimates was in Northwestern China, and the sampling locations were in the range 80–110° longitude (n = 10) and range 30–40° latitude (n = 8). Goats are economically significant animals in arid and semi-arid regions like Northwestern China because of their high adaptability (Abo-Shehada and Abo-Farieha, 2003; Chen, 2011). Our research showed that the prevalence of *Eimeria* spp. varies with precipitation levels. *Eimeria* spp. infection was more prevalent in places with moderate temperature and humidity (Mai et al., 2009; Keeton and Navarre, 2018). This point is consistent with the result in the season subgroup: *Eimeria* spp. prevalence in summer and autumn, with more rainfall, was higher than that in spring and winter, with less rainfall. Interestingly, in the temperature subgroup, the prevalence of *Eimeria* spp. infection in goats in China correlated negatively with temperature within a certain temperature range, although the difference was not significant. Infection by *Eimeria* spp. is considered to have no obvious seasonality (Hao, 2017). According to our results, we doubt whether there are more complex and hard-to-find connections between the *Eimeria* infection and seasons or not. At the same time, we found that many studies did not provide details of the sampling month, which also had a certain impact on our analysis of the seasons. When investigating goat coccidiosis, researchers should clarify the sampling month, because such details will help to analyze the effect of season and other climatic factors on goat coccidiosis. Notably, the results of both the altitude and humidity subgroups were generally high as most of the studies were before, 2006. This is consistent with the results of our research in the sampling year subgroup. However, in some areas, we only obtained a small number of studies, which might not reflect the true prevalence (Northeastern China = 2, Southern China = 1). This might also be one of the reasons for the insignificant differences.

For farmers who raise goats, high prevalence and highly pathogenic coccidia species will cause huge economic losses. According to our results, the prevalence of *E. alijevi*, *E. arloingi* and *E. christenseni*, were above 40%, and the prevalence of *E. caprina*, *E. hirci* and *E. ninakohlyakimovae* were all over 30%. Previous researches pointed out that when *E. ninakohlyakimovae* and *E. arloingi* are the main infective species, the fatality rate can reach 30% (Koudela and Boková, 1998). Moreover, we tried to conduct a subgroup analysis on the medication; however, no studies mentioned this information; therefore, we could not quantify it as a covariate for meta-analysis, although we believe that the correct use of anti-coccidial drugs might inhibit coccidiosis (Peek and Landman, 2003; Dang et al., 2019; Liu, 2019).

In the feeding model subgroup, the prevalence of infection in both feeding subgroups was close to 80%. A few weeks after newborn goats are infected, they can excrete millions of oocysts from their feces (Abo-Shehada and Abo-Farieha, 2003). When the oocysts are discharged into the farm with feces, the infection pressure caused by closed enclosure feeding is higher than that of grazing (Long and Joyner, 1984). We recommend that breeding farms should keep the breeding environment clean. Moreover, some disinfectants have important anticoccidial activity against oocysts and sporozoites of *Eimeria*, which is one of the key steps to control coccidiosis (López et al., 2019). Therefore, cleaning the feeding house with disinfectants might play an important role in the control of goat coccidiosis. However, the difference between subgroups was not significant, and further research is needed on the relationship between feeding methods and goat coccidiosis.

In the age subgroup, goats less than 1 year old had a higher prevalence, which might have been caused by resistance to *Eimeria* infection in adult animals that have been exposed to *Eimeria* previously (Carrau et al., 2018). *Eimeria* spp. has important economic significance for juvenile animals (Bawm et al., 2020). In juvenile animals, the occurrence of diarrhea can inhibit weight gain during the growth period (Daugschies and Najdrowski, 2005). Therefore, regarding coccidiosis, we suggest that more attention should be paid to younger animals. Furthermore, when adult animals are in a subclinical state, they can act as carriers of *Eimeria*, thus causing more goats to be infected (Carrau et al., 2016). Breeders should optimize the population structure and try to breed in groups, which might reduce the infection rate of *Eimeria* spp. In our study, female goats had a higher *Eimeria* infection prevalence than male goats. It might be explained that goat kids ingest oocysts attached to the udders of their dams finally lead to clinical signs (Kanyari, 1993). The kids then start excreting oocysts in feces from the second to the fourth weeks onwards and if these goat kids are kept with their mothers, the infection pressure can be high both in female goats and the kids (Ruiz and Molina, 2020).

Most of the 70 included studies used the saturated sodium chloride solution floating method [Float (NaCl), n = 50, and Float (C_12_H_22_O_11_), n = 14]. These are all traditional parasitic disease diagnosis methods that test for *Eimeria* oocysts. There were no significant differences between these methods in the reported prevalence. Our meta-regression analysis here suggested that detection methods were unlikely to be a significant source of heterogeneity in this analysis. There were only 5 studies with a score of 0–1, but 25 with a score of 2–3. Further research revealed that they did not clarify whether the sampling was random or not and the sampling method was not detailed, which caused them to lose some points. In addition, some risk factors were lacking that could not be analyzed, such as diarrhea. When investigating the prevalence of goat coccidiosis, researchers should collect and present more information.

This study had three limitations. First, before determining the search style, we tried different search styles in order to obtain a more comprehensive range of articles, however, there may be some omissions. Second, the number of studies from Southern and Northeastern China were few, which might have affected the analyses of the results from these regions. Third, the lack of some information (for example, whether the goats had diarrhea or not) will also affect the analysis results. However, we believe that this meta-analysis can reflect the true prevalence of *Eimeria* spp. infection in goats in China.

## Conclusion

Our analysis showed that the *Eimeria* spp. infection in goats is common in China. Most breeders do not pay attention to coccidiosis, resulting in a high overall prevalence. We suggest the development of different control strategies according to the geographical conditions of different regions. To further explore the susceptibility factors of goat coccidiosis, it is necessary to carry out epidemiological investigations in more areas and in detail.

## Data Availability Statement

The original contributions presented in the study are included in the article/**Supplementary Material**. Further inquiries can be directed to the corresponding authors.

## Author Contributions

RD and KS contributed to conception and design of this analysis. Q-LG provided the methodology. BZ, Z-YC, and YY collected the data and built the database. QW and Q-LG analyzed the results. N-CD prepared the manuscript. YC and J-ML revised the manuscript. All authors contributed to the article and approved the submitted version.

## Conflict of Interest

The authors declare that the research was conducted in the absence of any commercial or financial relationships that could be construed as a potential conflict of interest.

## Publisher’s Note

All claims expressed in this article are solely those of the authors and do not necessarily represent those of their affiliated organizations, or those of the publisher, the editors and the reviewers. Any product that may be evaluated in this article, or claim that may be made by its manufacturer, is not guaranteed or endorsed by the publisher.
