# Supplementary material for: Prevalence of Eimeria Spp. Among Goats in China: A Systematic Review and Meta-Analysis
Source: Front Cell Infect Microbiol. 2022 Mar 2;12:806085. doi: 10.3389/fcimb.2022.806085 (PMC8924409; doi:10.3389/fcimb.2022.806085)
Supplement: Supplementary file 1 [file Table_1.docx]

**Table S1** Items on the PRISMA checklist

| **Section/topic** | **#** | **Checklist item** | **Reported on page #** |
| --- | --- | --- | --- |
| **TITLE** |  |  |  |
| Title | 1 | Prevalence of *Eimeria* spp. among goats in China: A systematic review and meta-analysis | 1 |
| **ABSTRACT** |  |  |  |
| Structured summary | 2 | Provide a structured summary including, as applicable: background; objectives; data sources; study eligibility criteria, participants, and interventions; study appraisal and synthesis methods; results; limitations; conclusions and implications of key findings; systematic review registration number. | 2 |
| **INTRODUCTION** |  |  |  |
| Rationale | 3 | Describe the rationale for the review in the context of what is already known. | 3 |
| Objectives | 4 | Provide an explicit statement of questions being addressed with reference to participants, interventions, comparisons, outcomes, and study design (PICOS). | 3 |
| **METHODS** |  |  |  |
| Protocol and registration | 5 | Indicate if a review protocol exists, if and where it can be accessed (e.g., Web address), and, if available, provide registration information including registration number. | 3-6 |
| Eligibility criteria | 6 | Specify study characteristics (e.g., PICOS, length of follow-up) and report characteristics (e.g., years considered, language, publication status) used as criteria for eligibility, giving rationale. | 3-6 |
| Information sources | 7 | Describe all information sources (e.g., databases with dates of coverage, contact with study authors to identify additional studies) in the search and date last searched. | 3-6 |
| Search | 8 | Present full electronic search strategy for at least one database, including any limits used, such that it could be repeated. | 3-6 |
| Study selection | 9 | State the process for selecting studies (i.e., screening, eligibility, included in systematic review, and, if applicable, included in the meta-analysis). | 3-6 |
| Data collection process | 10 | Describe method of data extraction from reports (e.g., piloted forms, independently, in duplicate) and any processes for obtaining and confirming data from investigators. | 3-6 |
| Data items | 11 | List and define all variables for which data were sought (e.g., PICOS, funding sources) and any assumptions and simplifications made. | 3-6 |
| Risk of bias in individual studies | 12 | Describe methods used for assessing risk of bias of individual studies (including specification of whether this was done at the study or outcome level), and how this information is to be used in any data synthesis. | 3-6 |
| Summary measures | 13 | State the principal summary measures (e.g., risk ratio, difference in means). | 3-6 |
| Synthesis of results | 14 | Describe the methods of handling data and combining results of studies, if done, including measures of consistency (e.g., I^2^) for each meta-analysis. | 3-6 |
| Risk of bias across studies | 15 | Specify any assessment of risk of bias that may affect the cumulative evidence (e.g., publication bias, selective reporting within studies). | 3-6 |
| Additional analyses | 16 | Describe methods of additional analyses (e.g., sensitivity or subgroup analyses, meta-regression), if done, indicating which were pre-specified. | 3-6 |
| **RESULTS** |  |  |  |
| Study selection | 17 | Give numbers of studies screened, assessed for eligibility, and included in the review, with reasons for exclusions at each stage, ideally with a flow diagram. | 6-8, Figure 1 |
| Study characteristics | 18 | For each study, present characteristics for which data were extracted (e.g., study size, PICOS, follow-up period) and provide the citations. | 6-8, Tables 1 and S2 |
| Risk of bias within studies | 19 | Present data on risk of bias of each study and, if available, any outcome level assessment (see item 12). | 6-8, Figures 2 and 3, Figures S1-S10 and Tables S4 |
| Results of individual studies | 20 | For all outcomes considered (benefits or harms), present, for each study: (a) simple summary data for each intervention group (b) effect estimates and confidence intervals, ideally with a forest plot. | 6-8, Figure 2 |
| Synthesis of results | 21 | Present results of each meta-analysis done, including confidence intervals and measures of consistency. | 6-8, |
| Risk of bias across studies | 22 | Present results of any assessment of risk of bias across studies (see Item 15). | 6-8 |
| Additional analysis | 23 | Give results of additional analyses, if done (e.g., sensitivity or subgroup analyses, meta-regression [see Item 16]). | 6-8 |
| **DISCUSSION** |  |  |  |
| Summary of evidence | 24 | Summarize the main findings including the strength of evidence for each main outcome; consider their relevance to key groups (e.g., healthcare providers, users, and policy makers). | 8-12 |
| Limitations | 25 | Discuss limitations at study and outcome level (e.g., risk of bias), and at review-level (e.g., incomplete retrieval of identified research, reporting bias). | 12 |
| Conclusions | 26 | Provide a general interpretation of the results in the context of other evidence, and implications for future research. | 13 |
| **FUNDING** |  |  |  |
| Funding | 27 | Describe sources of funding for the systematic review and other support (e.g., supply of data); role of founders for the systematic review. | 13 |

*From:* Moher D, Liberati A, Tetzlaff J, Altman DG, The PRISMA Group (2009). Preferred Reporting Items for Systematic Reviews and Meta-Analyses: The PRISMA Statement. PLoS Med 6(6): e1000097. doi:10.1371/journal.pmed1000097

For more information, visit: **www.prisma-statement.org**

**Table S2** Studies included in the analysis

| **Study ID** | **Sampling time** | **Province** | **Method** | **Positive samples/**  **total samples** | **Quality score** | **Study Quality** | |
| --- | --- | --- | --- | --- | --- | --- | --- |
| **Central China** | | | | | | |  |
| Cao et al. 2015 | 2013.04-2014.08 | Henan | Float (C_12_H_22_O_11_) | 58/88 | 3 | Middle | |
| Liu. 2014 | 2011-2013 | Hunan | UN | 22/68 | 2 | Middle | |
| Zhu et al. 2013 | 2011.01-2012.02 | Henan | Float (C_12_H_22_O_11_) | 807/844 | 4 | High | |
| Qi. 2016 | 2014.9-12,2015.5-12,2016.01 | Hubei | Float (NaCl) | 485/668 | 3 | Middle | |
| Fan et al. 2014 | UN | Henan | UN | 150/150 | 2 | Middle | |
| Wang. 2017a | 2015.9-12 | Henan | Float (C_12_H_22_O_11_) | 65/109 | 4 | High | |
| Zhang et al. 1993 | UN | Henan, | Float (NaCl) | 102/292 | 2 | Middle | |
| Zhang et al. 2011 | 2010.03-05 | Henan, | Float (NaCl) | 74/203 | 3 | Middle | |
| Wei et al. 2012 | 2011.01-2012.02 | Henan | Float (C_12_H_22_O_11_) | 796/833 | 3 | Middle | |
| **East China** | | | | | | | |
| Liu et al. 2011 | 2010.04–2010.06 | Anhui | Float (NaCl) | 78/81 | 4 | High | |
| Wen et al. 2019 | 2019.03 | Shandong | Float (NaCl) | 26/32 | 2 | Middle | |
| Sun et al. 2010 | UN | Shandong | Float (NaCl) | 78/81 | 3 | Middle | |
| Li. 2018 | UN | Jiangsu | Float (C_12_H_22_O_11_) | 67/80 | 2 | Middle | |
| Kang et al. 2018 | UN | Jiangxi | Float (C_12_H_22_O_11_) | 213/312 | 3 | Middle | |
| Cai et al. 2018 | UN | Jiangsu | Float (NaCl) | 151/190 | 4 | High | |
| Yu et al. 2018 | UN | Shanghai | Float (C_12_H_22_O_11_) | 96/120 | 3 | Middle | |
| Ma. 2016 | 2015–2016 | Jiangsu | Float (NaCl) | 120/120 | 4 | High | |
| Lin et al. 2015 | 2015.01–2015.06 | Fujian | Float (NaCl) | 626/750 | 5 | High | |
| Gu et al. 2014 | 2012.10–2012.12 | Anhui | Float (C_12_H_22_O_11_) | 382/506 | 3 | Middle | |
| Zhu et al. 2013 | 2011.01–2012.02 | Anhui | Float (C_12_H_22_O_11_) | 77/80 | 4 | High | |
| Wei et al. 2012 | 2011.01–2012.02 | Anhui | Float (C_12_H_22_O_11_) | 77/80 | 4 | High | |
| Yang et al. 2012 | 2012.10–2013.02 | Anhui | Float (NaCl) | 80/90 | 5 | High | |
| Li et al. 2006 | 2004.04–06 | Anhui | Float (NaCl) | 94/100 | 4 | High | |
| Wang et al. 2000 | 1994/1997(11–12) | Shanghai | Float (NaCl) | 102/125 | 4 | High | |
| Chen et al. 1999 | 1991/1994(04–05) | Anhui | Float (NaCl) | 82/168 | 4 | High | |
| Wang. 2017a | 2015.9-12 | Anhui | Float (C_12_H_22_O_11_) | 176/574 | 4 | High | |
| Wang. 2017a | 2015.9-12 | Shandong | Float (C_12_H_22_O_11_) | 10/24 | 4 | High | |
| Wang. 2017a | 2015.9-12 | Jiangsu | Float (C_12_H_22_O_11_) | 2/74 | 4 | High | |
| Lin. 2020 | 2018.07-2019.12 | Fujian, | Float (NaCl) | 203/481 | 4 | High | |
| Wang et al. 1997 | 1996.7 | Jiangsu | Float (NaCl) | 52/54 | 3 | Middle | |
| Wang et al. 2020b | 2019.9-11 | Zhejiang | Float (NaCl) | 18/67 | 4 | High | |
| Tao et al. 2011 | UN | Anhui | UN | 22/24 | 1 | Low | |
| Xu et al. 2001 | 1998.04-1998.05 | Anhui | Float (NaCl) | 73/154 | 4 | High | |
| Peng et al. 1993 | UN | Jiangsu | Float (NaCl) | 62/85 | 1 | Low | |
| Dai et al. 1996 | 1994 | Jiangsu | Modified McMaster | 98/112 | 4 | High | |
| **North China** | | | | | | | |
| Zhang. 2018 | UN | Inner Mongolia | Float (NaCl) | 403/631 | 2 | Middle | |
| Wang et al. 2018 | UN | Inner Mongolia | Float (NaCl) | 102/140 | 2 | Middle | |
| Zhu et al. 2013 | 2011.01–2012.02 | Inner Mongolia | Float (C_12_H_22_O_11_) | 22/51 | 4 | High | |
| Wang et al. 1989 | 1986.9-1987.6 | Beijing | Float (NaCl) | 144/147 | 4 | High | |
| Wang et al. 2006 | 2002–2003 | Shanxi | Float (NaCl) | 106/112 | 5 | High | |
| **Northeast China** | | | | | | | |
| Lin et al. 2010 | UN | Heilongjiang | Float (NaCl) | 41/47 | 1 | Low | |
| Wang et al. 2010 | 2007.01–2009.06 | Heilongjiang | Float (NaCl) | 175/199 | 5 | High | |
| **Northwest China** | | | | | | | |
| Tu et al. 2016 | UN | Shaanxi | Float (NaCl) | 51/55 | 3 | Middle | |
| Feng et al. 2008 | UN | Shaanxi | Float (NaCl) | 454/1200 | 3 | Middle | |
| Feng et al. 2012 | 2009.10–2010.04 | Shaanxi | Float (NaCl) | 166/200 | 5 | High | |
| Yin et al. 2016 | 2014.12–2015.11 | Shaanxi | Float (NaCl) | 62/160 | 4 | High | |
| He et al. 2016 | 2014.03-2015.06 | Shaanxi | Float (NaCl) | 40/42 | 4 | High | |
| Cao et al. 2015 | 2013.04-2014.08 | Shaanxi | Float (C_12_H_22_O_11_) | 222/228 | 3 | Middle | |
| Zhu et al. 2013 | 2011.01-2012.02 | Qinghai | Float (C_12_H_22_O_11_) | 25/50 | 4 | High | |
| Liang et al. 2014 | 2019.5-7 | Shanxi | Float (NaCl) | 162/174 | 5 | High | |
| Yang et al. 1995 | 1991.6-1991.9 | Gansu | Float (C_12_H_22_O_11_) | 47/49 | 5 | High | |
| Wang et al. 2020a | UN | Shaanxi | Float (NaCl) | 11/55 | 3 | Middle | |
| Song et al. 2007 | UN | Shaanxi | Float (NaCl) | 188/188 | 4 | High | |
| Zhao et al. 2012 | 2010.11–2010.12 | Shaanxi | Float (NaCl) | 568/584 | 5 | High | |
| **South China** | | | | | | | |
| Wang. 2017b | UN | Guangxi | Float (NaCl) | 44/61 | 3 | Middle | |
| **Southwest China** | | | | | | | |
| Ma et al. 2018 | UN | Guizhou | Float (C_12_H_22_O_11_) | 503/532 | 1 | Low | |
| Zhang et al. 2014 | 2013.11–2014.01 | Sichuan | Float (NaCl) | 47/56 | 4 | High | |
| Tang et al. 2014 | 2013.09 | Guizhou | Float (NaCl) | 90/90 | 5 | High | |
| Chen et al. 2015 | 2011.08–2012.03 | Chongqing | Float (C_12_H_22_O_11_) | 285/301 | 5 | High | |
| Xia. 2018 | 2016.06–2016.09 | Tibet | Float (NaCl) | 40/48 | 4 | High | |
| Hao et al. 2018 | 2016.07–2016.08 | Sichuan | Modified McMaster | 456/464 | 4 | High | |
| Chang et al. 2017 | 2016.06–07 | Tibet | Float (C_12_H_22_O_11_) | 211/260 | 4 | High | |
| Ciren et al. 2016 | 2014–2016 | Tibet | Float (NaCl) | 19/207 | 4 | Hign | |
| Ruan et al. 2014 | 2012.08–2013.02 | Guizhou | Float (NaCl) | 328/339 | 5 | High | |
| Zhu et al. 2013 | 2011.01–2012.02 | Chongqing | Float (C_12_H_22_O_11_) | 16/18 | 4 | Hign | |
| Wei et al. 2012 | 2011.01–2012.02 | Chongqing | Float (C_12_H_22_O_11_) | 197/213 | 4 | High | |
| Xiao et al. 2012 | 2011.06 | Guizhou | Float (NaCl) | 87/150 | 5 | High | |
| OuYang et al. 2009 | 2008.03–2008.05 | Yunnan | Float (NaCl) | 6/31 | 3 | Middle | |
| Li et al. 1998 | 1995.04–1995.12 | Yunnan | Stauer's | 167/167 | 5 | High | |
| Yang et al. 1997 | UN | Yunan | Float (NaCl) | 101/114 | 3 | Middle | |
| Nie et al. 2001 | 1996.7 | Chongqing | Float (NaCl) | 102/128 | 2 | Middle | |
| Song et al. 1991 | 1988-1989 | Yunnan | Float (NaCl) | 557/565 | 4 | High | |
| Xia et al. 2015 | 2013.5-2014.4 | Tibet | Float (NaCl) | 1094/1200 | 4 | High | |
| Jiang et al. 2015 | 2014.03-2014.07 | Sichuan | Float (C_12_H_22_O_11_) | 96/101 | 3 | Middle | |
| Kuang. 2019 | 2015.5-2016.2 | Sichuan | Float (NaCl) | 324/7500 | 4 | High | |
| Chen. 2015 | 2011–2012 | Guizhou | Float (NaCl) | 1/230 | 3 | Middle | |
| **UN** | | | | | | | |
| Jiang et al. 1987 | 1986.09-1986.12 | UN | Float (MgSO₄) | 30/51 | 5 | High | |
| Liu et al. 2021 | 2019.4-7 | UN | UN | 284/352 | 2 | Middle | |
| Wei et al. 2013 | 2011.1-2012.11 | UN | Float (NaCl) | 1417/1489 | 4 | High | |
| Li et al. 2015 | 2011-2015 | UN | Float (NaCl) | 201/219 | 1 | Low | |

UN*: unclear.

**Table S3** Included studies and scores

| **Study No.** | **Reference ID** | **No. tested** | **No. positive** | **Prevalence** | **Random sampling or not** | **Detection method clearly or not** | **Sampled method detailedly or not** | **Sampled time clearly or not** | **Three or more risk factors or not** | **Score** | **Study Quality** |
| --- | --- | --- | --- | --- | --- | --- | --- | --- | --- | --- | --- |
| 1 | Zhang. 2018 | 631 | 403 | 63.87% | N | Y | N | N | Y | 2 | middle |
| 2 | Ma et al. 2018 | 532 | 503 | 94.55% | N | Y | N | N | N | 1 | low |
| 3 | Liu et al. 2011 | 81 | 78 | 96.30% | N | Y | Y | Y | Y | 4 | high |
| 4 | Zhang et al. 2014 | 56 | 47 | 83.93% | N | Y | Y | Y | Y | 4 | high |
| 5 | Tang et al. 2014 | 90 | 90 | 100.00% | Y | Y | Y | Y | Y | 5 | high |
| 6 | Wen et al. 2019 | 32 | 26 | 81.25% | N | Y | N | Y | N | 2 | middle |
| 7 | Tu et al. 2016 | 55 | 51 | 92.73% | N | Y | Y | N | Y | 3 | middle |
| 8 | Feng et al. 2008 | 1200 | 454 | 37.83% | Y | Y | Y | N | N | 3 | middle |
| 9 | Wei et al. 2013 | 1489 | 1417 | 95.16% | N | Y | Y | Y | Y | 4 | high |
| 10 | Li et al. 2015 | 219 | 201 | 91.78% | N | Y | N | N | N | 1 | low |
| 11 | Sun et al. 2010 | 81 | 78 | 96.30% | N | Y | Y | N | Y | 3 | middle |
| 12 | Feng et al. 2012 | 200 | 166 | 83.00% | Y | Y | Y | Y | Y | 5 | high |
| 13 | Chen et al. 2015 | 301 | 285 | 94.68% | Y | Y | Y | Y | Y | 5 | high |
| 14 | Xia. 2018 | 48 | 40 | 83.33% | Y | Y | N | Y | Y | 4 | high |
| 15 | Wang et al. 2018 | 140 | 102 | 72.86% | Y | Y | Y | N | N | 3 | middle |
| 16 | Li. 2018 | 80 | 67 | 83.75% | N | Y | N | N | Y | 2 | middle |
| 17 | Kang et al. 2018 | 312 | 213 | 68.27% | Y | Y | Y | N | N | 3 | middle |
| 18 | Hao et al. 2018 | 464 | 456 | 98.28% | N | Y | Y | Y | Y | 4 | high |
| 19 | Cai et al. 2018 | 190 | 151 | 79.47% | Y | Y | Y | N | Y | 4 | high |
| 20 | Yu et al. 2017 | 120 | 96 | 80.00% | N | Y | Y | N | Y | 3 | middle |
| 21 | Wang. 2017b | 61 | 44 | 72.13% | Y | Y | Y | N | N | 3 | middle |
| 22 | Qi. 2016 | 668 | 485 | 72.60% | N | Y | N | Y | Y | 3 | middle |
| 23 | Chang et al. 2017 | 260 | 211 | 81.15% | Y | Y | N | Y | Y | 4 | high |
| 24 | Yin et al. 2016 | 160 | 62 | 38.75% | Y | Y | N | Y | Y | 4 | high |
| 25 | Ma. 2016 | 120 | 120 | 100.00% | Y | Y | N | Y | Y | 4 | high |
| 26 | He et al. 2016 | 42 | 40 | 95.24% | Y | Y | N | Y | Y | 4 | high |
| 27 | CiRen et al. 2016 | 207 | 19 | 9.18% | Y | Y | N | Y | Y | 4 | high |
| 28 | Lin et al. 2015 | 750 | 626 | 83.47% | Y | Y | Y | Y | Y | 5 | high |
| 29 | Cao et al. 2015 | 316 | 280 | 88.61% | N | Y | N | Y | Y | 3 | middle |
| 30 | Ruan et al. 2014 | 339 | 328 | 96.76% | Y | Y | Y | Y | Y | 5 | high |
| 31 | Liu. 2014 | 68 | 22 | 32.35% | Y | N | N | Y | N | 2 | middle |
| 32 | Gu et al. 2014 | 506 | 382 | 75.49% | N | Y | N | Y | Y | 3 | middle |
| 33 | Zhu et al. 2013 | 1043 | 947 | 90.80% | N | Y | Y | Y | Y | 4 | high |
| 34 | Wei et al. 2012 | 1126 | 1070 | 95.03% | N | Y | Y | Y | Y | 4 | high |
| 35 | Yang et al. 2012 | 90 | 80 | 88.89% | Y | Y | Y | Y | Y | 5 | high |
| 36 | Xiao et al. 2012 | 150 | 87 | 58.00% | Y | Y | Y | Y | Y | 5 | high |
| 37 | Lin et al. 2010 | 47 | 41 | 87.23% | N | Y | N | N | N | 1 | low |
| 38 | OuYang et al. 2009 | 31 | 6 | 19.35% | N | Y | N | Y | Y | 3 | middle |
| 39 | Wang et al. 2006 | 112 | 106 | 94.64% | Y | Y | Y | Y | Y | 5 | high |
| 40 | Li et al. 2006 | 100 | 94 | 94.00% | N | Y | Y | Y | Y | 4 | high |
| 41 | Wang et al. 2000 | 125 | 102 | 81.60% | Y | Y | N | Y | Y | 4 | high |
| 42 | Chen et al. 1999 | 168 | 82 | 48.81% | N | Y | Y | Y | Y | 4 | high |
| 43 | Li et al. 1998 | 167 | 167 | 100.00% | Y | Y | Y | Y | Y | 5 | high |
| 44 | Yang et al. 1997 | 114 | 101 | 88.60% | N | Y | Y | N | Y | 3 | middle |
| 45 | Dai et al. 1996 | 112 | 98 | 87.50% | Y | Y | N | Y | Y | 4 | high |
| 46 | Chen. 2015 | 230 | 1 | 0.43% | N | Y | N | Y | Y | 3 | middle |
| 47 | Zhao et al. 2012 | 584 | 568 | 97.26% | Y | Y | Y | Y | Y | 5 | high |
| 48 | Wang et al. 2010 | 199 | 175 | 87.94% | Y | Y | Y | Y | Y | 5 | high |
| 49 | Wang et al. 1989 | 147 | 144 | 97.96% | Y | Y | Y | N | Y | 4 | high |
| 50 | Fan et al. 2014 | 150 | 150 | 100.00% | Y | N | N | N | Y | 2 | middle |
| 51 | Liang et al. 2014 | 174 | 162 | 93.10% | Y | Y | Y | Y | Y | 5 | high |
| 52 | Wang. 2017a | 781 | 253 | 32.39% | N | Y | Y | Y | Y | 4 | high |
| 53 | Lin. 2020 | 481 | 203 | 42.20% | N | Y | Y | Y | Y | 4 | high |
| 54 | Yang et al. 1995 | 49 | 47 | 95.92% | Y | Y | Y | Y | Y | 5 | high |
| 55 | Nie et al. 2001 | 128 | 102 | 79.69% | N | Y | N | N | Y | 2 | middle |
| 56 | Wang et al. 1997 | 54 | 52 | 96.30% | N | Y | N | Y | Y | 3 | middle |
| 57 | Wang et al. 2020b | 67 | 18 | 26.87% | Y | Y | N | Y | Y | 4 | high |
| 58 | Wang et al. 2020a | 55 | 11 | 20.00% | Y | Y | N | N | Y | 3 | middle |
| 59 | Song et al. 2007 | 188 | 188 | 100.00% | Y | Y | Y | N | Y | 4 | high |
| 60 | Song et al. 1991 | 565 | 557 | 98.58% | N | Y | Y | Y | Y | 4 | high |
| 61 | Xia et al. 2015 | 1200 | 1094 | 91.17% | N | Y | Y | Y | Y | 4 | high |
| 62 | Jiang et al. 2015 | 101 | 96 | 95.05% | N | Y | N | Y | Y | 3 | middle |
| 63 | Tao et al. 2011 | 24 | 22 | 91.67% | N | N | N | N | N | 1 | low |
| 64 | Xu et al. 2001 | 154 | 73 | 47.40% | N | Y | Y | Y | Y | 4 | high |
| 65 | Jiang et al. 1987 | 51 | 30 | 58.82% | Y | Y | Y | Y | Y | 5 | high |
| 66 | Liu et al. 2021 | 352 | 284 | 80.68% | N | N | N | Y | Y | 2 | middle |
| 67 | Kuang. (2019) | 7500 | 324 | 4.32% | N | Y | Y | Y | Y | 4 | high |
| 68 | Zhang et al. 1993 | 292 | 102 | 34.93% | N | N | Y | N | Y | 2 | middle |
| 69 | Peng et al. 1993 | 85 | 62 | 72.94% | N | N | N | N | Y | 1 | low |
| 70 | Zhang et al. 2011 | 203 | 74 | 36.45% | N | N | Y | Y | Y | 3 | middle |

**Reference**

1. Zhang TZ. Epidemiological survey of coccidiosis in central production area of Arbas White Cashmere Goat. *Inner Mongolia Agricultural University*. (2018). (In Chinese)
2. Ma JP, Wu DY, Song DR, Zhang QD, Zhou DR, Peng H, et al. Investigation and Analysis of Coccidia Infection in Large-scale Sheep Farms in Alpine Regions. *Heilongjiang Anim Sci Vet Med*. (2018) 04: 110-112+239. (In Chinese)
3. Liu J, Ge L. Investigation on Coccidia Infection in Sheep Farm in Hefei Province. C*hi J Anim Husb Vet Med* (2011) 27 (02): 77-80. (In Chinese)
4. Zhang WL, Hao GY, Luo QH. Infect Status of Gastronintestinal Parasites in Huili Black Gost. *Guizhou Agr Sci*. (2014) 42: 107-110. (In Chinese)
5. Tang JG, Li HS, Xiao FP. Detection and control of parasites on grazing goats on artificial grassland. *Chin J Anim Husb Vet Med*. (2014) 30: 78. (In Chinese)
6. Wen HY, Dong BX, Kong N, Ge SH, Jiang LL. Detection of intestinal parasites in some free-range Jining green goats in Heze City, Shandong Province. *China Anim Health Inspection*. (2019) 36: 24-27+35. (In Chinese)
7. Tu YQ, Jian YL, Gao YA. Investigation and identification of coccidiosis of Jianyang big-eared goat in a sheep farm in Shaanxi Province. *Prog Vet Med*. (2016) 37: 132-135. (In Chinese)
8. Feng P, Yu SK, Qu L, Ma F, Wang F. Investigation on Digestive Tract Parasites of Yulin Goat in Shaanxi Province. *Prog Vet Med*. (2008) 06: 117-119. (In Chinese)
9. Wei JJ. Epidemiological investigation of sheep coccidiosis species and piriformis in parts of China. *Henan Agricultural University*. (2013). (In Chinese)
10. Li J. Investigation on four pathogenic infections of sheep in three regions of China. *Henan Agricultural University*. (2015). (In Chinese)
11. Sun ZH, Zhang XB, Liu J, Ding JP. Investigation of Goat Coccidiosis Infection in Sheep Farm. *Modern Agrl Sci Technol*. (2010) 18: 297-298+301. (In Chinese)
12. Feng P, Bian ZT, Qu L. Investigation on the infection of digestive tract parasites in northern Shaanxi white cashmere goats in Yulin. *Heilongjiang Anim Sci Vet Med.* (2012) 09: 117-118. (In Chinese)
13. Chen J, He W, Ren HX, Gao LF, Ning CS. Investigation on intestinal parasite infection of goats in some sheep farms in Chongqing. *J Southwest Univ (Nat Sci Edit)*. (2015) 37: 71-75. (In Chinese)
14. Xia CY, Liu JZ, Song TZ, Weng HB, Zhuo G, DanQu LM, et al. Investigation of Coccidiosis in Lambs in Parts of Tibet. *China Herbivore Sci*. (2018) 38: 36-39. (In Chinese)
15. Wang J, Wang Y, HuGe JLT, SuYa LT, Tuo NL. Epidemiological Investigation on Parasitic of Arbas White Cashmere Cashmere Goats in Otog Banner and Serum Biochemical Parameters Analysis of the Goats with Diarrhea. *Anim Husbandry and Feed Science*. (2018) 39(4): 106-109. (In Chinese)
16. Li YM. Investigation on species and infection of goat coccidia in Rudong area of Jiangsu Province. *Graziery Vet Sci*. (2018) 38: 10-11. (In Chinese)
17. Kang JW, Zheng WY, Wu QQ, Wen YT, Yuan B, Liu B. Investigation on Intestinal Parasite Infection in Goats in Western Jiangxi. *Heilongjiang Anim Sci Vet Med.* (2018) 10: 103-105, 244-245. (In Chinese)
18. Hao GY, Yang YD, Xu R, Deng P. Investigation of caprine coccidial infection in Yanbian county. *Anim Hus Vet Med*. (2018) 50(08): 69-72. (In Chinese)
19. Cai WM, Gao Y, Liu DD, Cheng DR, Xing H, Yang Al, et al. Epidemiology of gastrointestinal parasite and Toxoplasma gondii of goats in the Yangzhou area. *Anim Husb Vet Med*. (2018) 50: 108-112. (In Chinese)
20. Yu XX, Xing T, Li JG. Investigation on Coccidian Species and Infection Situation of Goats in Port-surrounding Area of Shanghai City. *China Anim Health Inspection.* (2017) 34: 13-15. (In Chinese)
21. Wang HQ. Investigation and Analysis of Sheep Parasites in Wuxuan County. *J Guangxi Agri*. (2017) 32: 33-36. (In Chinese)
22. Qi MW. Investigation and species identification of goat coccidia infection in Hubei Province. *Hubei: Huazhong Agricultural University*. (2016). (In Chinese)
23. Chang YK, Wu YY, Zheng SJ, Wang L, Li DF, Li JK, et al. InVestigation of Coccidial Infbction and Species in Goats in Xaitongmoin, Tibet. *Chin J Vet Med*. (2017) 53: 9-12. (In Chinese)
24. Yin HK, Wang XZ, Wang ZL, Zhang SG, Ren BH, Zhang SL. Investigation and Comprehensive Prevention of Meat Goat Parasitic Disease in Linyou County. *J Anim Sci Vet Med*. (2016) 35: 84-87. (In Chinese)
25. Ma F. Epidemiological survey of intestinal parasites in livestock from 2015 to 2016 in Si County, Anhui Province. *Anhui Agricultural University*. (2016). (In Chinese)
26. He SJ, He XL, Yu SK. Investigation on Coccidial Species and Infection Status of Goats in a Goat Farm of Yangling. *Prog Vet Med*. (2016) 37: 133-136. (In Chinese)
27. CiRem DJ, Qu J, Dan Z, Ba D, Da W, YiXi DJ. Cause Investigation of the Death of Northwest Tibet Lambs. *Anim Husb Feed Sci.* (2016) 37: 98-100,101. (In Chinese)
28. Lin L, Jiang B, Wu SH, Zhang SZ, Li CS, Lin S, et al. The Investigation of Coccidial Infection of Goat under Different Conditions in Fujian. *Fujian J Agr Sci*. (2015) 7: 642-647. (In Chinese)
29. Cao SX, Zhang ZM, Cui YY, Wang JH, Lv YL, Li D, et al. Survey on the Prevalence of Intestinal Parasites in Dairy Goats in Partial Regions of China. *J Henan Agr Sci.* (2015) 44: 146-149. (In Chinese)
30. Ruan ZX, Long DM, Liu J, He XF, Liu J, Wang YH, et al. Applicadon of Conlputer Technology in Identi6ca60n of Goat Coccidiosis Species. *China Herbivore Sci* (2014) 34: 39-42. (In Chinese)
31. Liu BC. Investigation on the pathogen of goat parasites in Xiangxi Autonomous Prefecture. *Hunan J Anim Sci Vet Med*. (2014) 5: 26-28. (In Chinese)
32. Gu YF, Li WC, Wang LK, Li Y, Li L, Zhu XH, et al. Investigation of intestinal parasite infection in goats in Anhui Province. *Anim Husb Vet Med*. (2014) 46: 100-103. (In Chinese)
33. Zhu D, Lv YL, Li MJ, Zhang ZJ, Jian FC, Song D, et al. Investigation of intestinal parasite infection in goats in some regions of China. *China Herbivore Sci*. (2013) 33: 43-46. (In Chinese)
34. Wei JJ, Zhang XD, Zhang ZJ, Jian FC, Qi M, Zhang FF, et al. Investigation on Coccidia Prevalence and Species in Goats in Partial Areas of China. *China Herbivore Sci*. (2012) 32: 34-38. (In Chinese)
35. Yang ZP, Li S, Li PY. Investigation on the species and infection of goat coccidia in Hefei area. *Agr Technol Serv*. (2012) 29(12): 1328. (In Chinese)
36. Xiao FP, Zhou SX, Liu H, Xu J, Xu HZ, Dong FX, et al. Investigation and control of goat's digestive tract parasite infection. *China Anim Husb Vet Med.* （2012） 39: 195-197. (In Chinese)
37. Lin R, Li JH, Gao JF, Wang CR. Species survey and morphological description of sheep and goat coccidia in parts of Heilongjiang Province. *Modern Anim Husb Sci Technol*. (2010) 38: 209-10. (In Chinese)
38. OuYang XF, Yang JF, He XJ, Long W. Investigation of gastrointestinal parasites in goats in a slaughterhouse in the northern suburb of Kunming. *Yunnan J Anim Sci Vet Med*. (2009) 38: 103-6. (In Chinese)
39. Wang TF, Wang J. Survery on Goat Coccidium Forms and Infection in Xiangyuan County. *J Shanxi Agr Sci*. (2006) 34: 83-4. (In Chinese)
40. Li PY, Wang JH, Zhou YZ, Zhao CC. Investigation on the species and infection of goat coccidia in Hefei area. *Anim Husb Vet Med*. (2006) 38: 20-3. (In Chinese)
41. Wang WD, Chen DM, Lv RQ, Fu XP, Pan HJ, Shen YL. Investigation on Goat Coccidia in Nanjing. *Chin J Anim Infect Dis*. (2000) 8: 26-7. (In Chinese)
42. Chen H, Zhang H, Gu YF, Chen DM, Ren JR, Liu ZH, Shen YL. Investigation of goat coccidia species in Bengbu area. *Chin J Anim Infect Dis*. (1999) 7: 28-30. (In Chinese)
43. Li JG, Huang WZ, Xu SX, Tian BP, Hou YK, Yang XC, et al. Investigation on Spring Lamb Coccidiosis Infection in Zhenyuan County. *Chin J Anim Infect Dis*. (1998) 6: 33-4. (In Chinese)
44. Yang YZ, Zeng L. Investigation on Goat Coccidium Species in the Northern Suburb of Kunming. *J Yunnan Agr U (Nat Sci)*. (1997) 12: 58-61. (In Chinese)
45. Dai YB, Tao H, Fu AQ, Tao JP. Investigation of Goat Coccidiosis Infection and Single Oocyst Infection Test in Yancheng. *Jiangsu Agr Sci*. (1996) 24: 57-59. (In Chinese)
46. Chen ZH. Investigation Report on Parasite Infection in Goats of Changshun County. *Agr Tech Serv*. (2015) 32: 172 -174. (In Chinese)
47. Zhao GH, Lei LH, Shang CC, et al. High prevalence of Eimeria infection in dairy goats in Shaanxi province, northwestern China. *Trop Anim Health Prod*. (2012) 44: 943-946. doi:10.1007/s11250-011-9997-8
48. Wang CR, Xiao JY, Chen AH, et al. Prevalence of coccidial infection in sheep and goats in northeastern China. *Vet Parasitol*. (2010) 174: 213-217. doi:10.1016/j.vetpar.2010.08.026
49. Wang M, Su C. Investigation of goat coccidia species in Beijing. *Chin J Vet Med*. (1989) 37: 4-6+15. (In Chinese)
50. Fan SH, Wang YL. Epidemiological investigation of intestinal parasites in Huai goats. *Heilongjiang Anim Husb Vet*. (2014) 57: 67-68. (In Chinese)
51. Liang HL, Sun QW, Zhu B, Liu J. Investigation on parasitic infection in goat digestive tract in Yulin, Shaanxi. *Foreign Anim Husb (Pigs Birds)*. (2019) 39: 53-56. (In Chinese)
52. Wang K. Epidemiological survey of sheep intestinal parasitic diseases in Anhui and surrounding areas. *Anhui Science and Technology University.* (2017). (In Chinese)
53. Lin CY. Investigation on parasitic infection in goat digestive tract in JuKou Town, Jianyang District, Nanping City. *Fujian Agriculture and Forestry University*. (2020). (In Chinese)
54. Yang SX., Lv GQ, Yu QL. Investigation of coccidiosis infection in goat lambs of the year in the original village of Liucun, Qingyang County. *Chin J Anim Infect Dis*. (1995) 3: 41-43. (In Chinese)
55. Nie K, Yuan CG, Chen H. Investigation on the species of goat coccidia in Rongchang area of Chongqing. *J Teach Educ*. (2001) 15: 28-32. (In Chinese)
56. Wang XZ, Yang XY, Xu GL, Sun LH, Wu ZR. Investigation on Coccidia Infection and Species of White Goats in Danyang City. *Chin J Anim Infect Dis*. (1997) 5: 25-26. (In Chinese)
57. Wang M, Luo HQ, Jian YL. Epidemiological investigation of parasites in the digestive tract of black goats in Wenzhou. *Livest Poultry Ind*. (2020) 31:8+10. (In Chinese)
58. Wang N, Li C. Epidemiological investigation of parasites in the digestive tract of Shaanbei white cashmere goats in Yulin City. *Farmers Consult*. (2020) 38: 147. (In Chinese)
59. Song JK, Yu SK, Yuan C. Investigation and Study on parasitic species in a sheep farm in Yongshou County, Shaanxi. *Prog Anim Med*. (2007) 28: 107-8. (In Chinese)
60. Song XL, Lin YY, Tu Y, Zuo YG. Investigation on coccidia species of mountain sheep in Yunnan Province. *Chin Vet Sci Tech*. (1991) 21: 17-21. (In Chinese)
61. Xia CY, Liu JZ, Song TZ, Feng J, Yuan ZJ, Ban D, et al. Investigation of goat coccidia infection in Nimu County, Tibet. *Prog Anim Med*. (2015) 36: 120-3. (In Chinese)
62. Jiang HL, Hao WC. Investigation of Goat Parasite Infection in Yibin City. *Chin Anim Husb Vet Digest*. (2015) 31: 118. (In Chinese)
63. Tao Y, Li PY, Tao XP, Ju SC, Liu Y. Investigation on Goat Parasites in Shushan District of Hefei City. *J Anhui Agr U*. (2001) 45: 161-163. (In Chinese)
64. Xu ZK, Sheng WY. Investigation on Coccidia Infection and Species of Goats in Quanjiao County. *J Anhui Agr Sci*. (2001) 41: 546-547. (In Chinese)
65. Jiang XS, Zhu HQ. A study of coccidia in sheep and goats. *J Southwest Minzu U (Nat Sci Edition)*. (1987) 13: 38-43. (In Chinese)
66. Liu LK, et al. Investigation of parasite infections in the digestive tract of sheep and identification of coccidia species in some areas of my country. *Anim Husb Vet Med*. (2021) 53: 117-123. (In Chinese)
67. Kuang SY. Investigation on the internal parasite infection of black goats in a certain county of Sichuan Province. *Sichuan Agricultural University*. (2019). (In Chinese)
68. Zhang L, Zhou JX, Ren TQ, Hou ZA, Wang SR, Feng XR. Investigation on species and epidemiology of coccidia in sheep and goats in Henan Province. *Henan J Anim Husb Vet Med*. (1993) 16: 24-25. (In Chinese)
69. Peng DW, Yang TG, Kuang CL, Xu ZQ, Han JL, Guo JS, et al. A survey of coccidia species in goats in Tai County, Jiangsu. *Anim Husb Vet Med*. (1993) 44: 155-156. (In Chinese)
70. Zhang L, Kong ZY, Zheng J, Yang HM, Yan JF, Xia K. Investigation on species and infection status of goat coccidia in Luoyang suburb. *Anim Husb Vet Med*. (2011) 43: 108-10. (In Chinese)

**Table S4** The formulas for PFT

| t = arcsin (sqrt (r/(n + 1))) + arcsin (sqrt ((r + 1)/(n + 1))) |
| --- |
| se(t) = sqrt(1/(n + 0.5)) |
| p = (sin(t/2))^2^ |

Note: t: transformed prevalence; n = sample size; r = positive number; se = standard error.

**Table S5.** The code in R for this meta-analysis.

| Logarithmic conversion (PNL) | rate<-transform [m1, log=log(event/n)];  shapiro.test(rate$log) |
| --- | --- |
| Logit transformation (PLOGIT) | rate<-transform{m1, logit=log[(event/n)/(1-event/n)]};  shapiro.test(rate$logit) |
| Arcsine transformation (PAS) | rate<-transform{m1, arcsin.size=asin[sqrt(event/(n+1))]};  shapiro.test(rate$arcsin) |
| Double-arcsine transformation (PFT) | rate<-transform{m1,darcsin=0.5*[asin(sqrt(event/(n+1)))+asin((sqrt(event+1)/(n+1)))]};  shapiro.test(rate$darcsin) |
| No transformation (PRAW) | rate<-transform[m1, r= event/n];  shapiro.test(rate$r) |
| Forest plots | forest [meta1, xlim=c(-0.2, 0.8)] |
| Funnel chart | funnel (meta1) |
| Egger's test | metabias (meta1, method="linreg") |
| The sensitivity analysis | metainf (meta1, pooled = "random") forest (metainf (meta1, pooled = "random"), xlim=c(0, 0.3)) |
| Subgroup analysis | meta1<-metaprop(event, n, study, data=rate, sm="PLN", incr=0.5, allincr=TRUE, addincr=FALSE, title="", byvar= subgroup title, print.byvar=TRUE) |
| Meta-regression analysis | metareg (meta1, ~covariate title) |

**Table S6** Egger’s test for publication bias

| slope | bias | se. bias | t | df | *P*-value |
| --- | --- | --- | --- | --- | --- |
| 0.5403 | 15.7314 | 3.3885 | 4.643 | 68 | 1.622e-05 |

**Table S7** Estimates of *Eimeria* spp. infection in goats in China

| Region | Estimated number of goats in various regions of China* | Prevalence of *Eimeria* spp. infection of goats in various regions of China | Estimated number of goats with *Eimeria* spp. infection |
| --- | --- | --- | --- |
| Northwestern China | 19,622,000 | 80.7% (95%CI: 59.36-95.42) | 15,834,954 (11,655,468–18,719,388) |
| Southwestern China | 36,521,000 | 78.4% (95%CI: 50.75-96.62) | 28,632,464 (18,534,408–35,286,590) |
| Northeastern China | 6,467,000 | 88.0% (95%CI: 83.54-91.86) | 5,690,960 (5,402,532–5,940,586) |
| Central China | 26,181,000 | 70.9% (95%CI: 50.57-87.63) | 18,562,329 (13,239,732–22,942,410) |
| Eastern China | 20,692,000 | 79.8% (95%CI: 69.78-88.21) | 16,512,216 (14,443,016–18,250,344) |
| North China | 24,914,000 | 78.4% (95%CI: 57.25-93.78) | 19,532,576 (14,263,265–23,364,349) |
| South China | 3,840,000 | 72.1% (95%CI: 60.13-82.75) | 2,768,640 (2,308,992–3,177,600) |
| Total | 138,237,000 | 78.7% (95%CI: 68.15-87.67) | 108,792,519 (94,277,634–121,233,849) |

*Estimates of the number of goats in each region were obtained from 2018 data of the Chinese Animal Husbandry and Veterinary Yearbook report.

**Figure S1** Egger’s test for publication bias

0

50

100

150

0

20

40

60

80

100

Inverse of standard error

Standardised treatment effect (z-score)

**Figure S2.** Funnel plot with a trim and fill analysis for the publication bias test

0.0

0.5

1.0

1.5

0.10

0.08

0.06

0.04

0.02

0.00

Freeman-Tukey Double Arcsine Transformed Proportion

Standard Error

**Figure S3** Funnel plot with pseudo 95% confidence intervals for the examination of publication bias by region

0.5

1.0

1.5

0.12

0.10

0.08

0.06

0.04

0.02

0.00

Freeman-Tukey Double Arcsine Transformed Proportion

Standard Error

**Figure S4** Funnel plot with pseudo 95% confidence intervals for the examination of publication bias by sampling year

0.5

1.0

1.5

0.08

0.06

0.04

0.02

0.00

Freeman-Tukey Double Arcsine Transformed Proportion

Standard Error

**Figure S5** Funnel plot with pseudo 95% confidence intervals for the examination of publication bias by detection methods

0.5

1.0

1.5

0.08

0.06

0.04

0.02

0.00

Freeman-Tukey Double Arcsine Transformed Proportion

Standard Error

**Figure S6** Funnel plot with pseudo 95% confidence intervals for the examination of publication bias by feeding model

0.5

1.0

1.5

0.10

0.08

0.06

0.04

0.02

0.00

Freeman-Tukey Double Arcsine Transformed Proportion

Standard Error

**Figure S7** Funnel plot with pseudo 95% confidence intervals for the examination of publication bias by age

0.4

0.6

0.8

1.0

1.2

1.4

1.6

0.15

0.10

0.05

0.00

Freeman-Tukey Double Arcsine Transformed Proportion

Standard Error

**Figure S8** Funnel plot with pseudo 95% confidence intervals for the examination of publication bias by gender

0.6

0.8

1.0

1.2

1.4

0.12

0.10

0.08

0.06

0.04

0.02

0.00

Freeman-Tukey Double Arcsine Transformed Proportion

Standard Error

**Figure S9** Funnel plot with pseudo 95% confidence intervals for the examination of publication bias by season

0.5

1.0

1.5

0.12

0.10

0.08

0.06

0.04

0.02

0.00

Freeman-Tukey Double Arcsine Transformed Proportion

Standard Error

**Figure S10** Funnel plot with pseudo 95% confidence intervals for the examination of publication bias by quality level

0.5

1.0

1.5

0.10

0.08

0.06

0.04

0.02

0.00

Freeman-Tukey Double Arcsine Transformed Proportion

Standard Error
